# PPAR Alpha as a Metabolic Modulator of the Liver: Role in the Pathogenesis of Nonalcoholic Steatohepatitis (NASH)

**DOI:** 10.3390/biology11050792

**Published:** 2022-05-23

**Authors:** Simona Todisco, Anna Santarsiero, Paolo Convertini, Giulio De Stefano, Michele Gilio, Vito Iacobazzi, Vittoria Infantino

**Affiliations:** 1 Department of Science, University of Basilicata, Viale dell’Ateneo Lucano 10, 85100 Potenza, Italy; simona.todisco@unibas.it (S.T.); santarsieroanna90@gmail.com (A.S.); paolo.convertini@gmail.com (P.C.); 2 Department of Infectious Diseases, San Carlo Hospital, Via Potito Petrone, 85100 Potenza, Italy; drgiuliodestefano@gmail.com (G.D.S.); michelegilio@gmail.com (M.G.); 3 Department of Biosciences, Biotechnologies and Biopharmaceutics, University of Bari, Via Orabona 4, 70125 Bari, Italy; vito.iacobazzi1@gmail.com

**Keywords:** PPAR alpha, NASH, metabolism, liver, inflammation, gene regulation

## Abstract

**Simple Summary:**

In the context of liver disease, one of the more growing public health problems is the transition from simple steatosis to non-alcoholic steatohepatitis. Profound metabolic dysregulations linked to inflammation and hepatic injury are features of non-alcoholic steatohepatitis. Since the peroxisomal-proliferator-activated receptor alpha has long been considered one of the key transcriptional factors in hepatic metabolism, its role in the pathogenesis of non-alcoholic steatohepatitis is discussed in this review.

**Abstract:**

The strong relationship between metabolic alterations and non-alcoholic steatohepatitis (NASH) suggests a pathogenic interplay. However, many aspects have not yet been fully clarified. Nowadays, NASH is becoming the main cause of liver-associated morbidity and mortality. Therefore, an effort to understand the mechanisms underlying the pathogenesis of NASH is critical. Among the nuclear receptor transcription factors, peroxisome-proliferator-activated receptor alpha (PPARα) is highly expressed in the liver, where it works as a pivotal transcriptional regulator of the intermediary metabolism. In this context, PPARα’s function in regulating the lipid metabolism is essential for proper liver functioning. Here, we review metabolic liver genes under the control of PPARα and discuss how this aspect can impact the inflammatory condition and pathogenesis of NASH.

## 1. Introduction

Among the broad spectrum of nonalcoholic fatty liver diseases (NAFLD), nonalcoholic steatohepatitis (NASH) represents a severe condition characterized by the presence of a chronic inflammation and injury of the hepatic parenchyma—in addition to the hepatic steatosis—in patients with no history of alcohol abuse [1]. NAFLD has been described as a metabolic dysfunction of the liver often linked to insulin resistance and induced hepatic lipogenesis. Although NAFLD is a benign condition, it is becoming a global health problem with an incidence of about 25%, a part of which (10–30%) may be classified as NASH [2]. Moreover, a subset of patients with NASH progress to liver fibrosis, leading to cirrhosis and hepatocellular carcinoma (HCC), thus needing liver transplantation. Since NASH is becoming the main cause of liver-associated morbidity and mortality [3,4,5,6], the mechanism by which steatosis progression leads to NASH is one of the most studied aspects of the liver’s pathophysiology.

An important factor in the onset of early NAFLD and then NASH is the modulatory role played by the peroxisome-proliferator-activated receptor alpha (PPARα). PPARα belongs to the nuclear receptor transcription factor family (PPARs), which include three isoforms: PPARα, PPARβ/δ and PPARγ [7]. PPARs show a different expression pattern and set of functions [8]. While PPARβ/δ is expressed in the skeletal muscle and PPARγ in the adipose tissue, PPARα is particularly abundant in the liver [9], but it is expressed and active in many other tissues, including skeletal muscles, adipose tissues, gut, kidneys and the heart, which all contribute to fatty acid homeostasis [10]. Since its discovery, the role of PPARα has evolved from an intracellular receptor for peroxisome proliferators into one of the most important transcriptional regulators of the intermediary metabolism and a relevant player in the pathogenesis of numerous diseases [11]. PPARα is the master regulator of lipid metabolism via regulation of numerous genes involved in fatty acid uptake and activation, mitochondrial and peroxisomal fatty acid oxidation, ketogenesis, triglyceride turnover, lipid droplet biology, gluconeogenesis and bile synthesis/secretion. Moreover, other involvements, such as glucose metabolism and homeostasis, control of glycerol for gluconeogenesis [12], cell differentiation and proliferation, cancer, and pro-inflammatory and anti-inflammatory activity [13] can be ascribed to PPARα [14]. Different genes of amino acids’ metabolism, such as transamination and deamination, are down regulated by PPARα [15]. Furthermore, PPARα regulates the expression of the fibroblast growth factor 21 (FGF21) during starvation [16] and helps to repress the acute-phase response and inflammation in the liver [17]. A liver fatty acid homeostasis impairment was observed in a PPARα knockout mouse model leading to hepatic lipid accumulation and hypercholesterolemia during aging [18] and an increased amount of circulating free fatty acids [19]. These conditions cause an increase in the production of reactive oxygen species (ROS), strictly related to high levels of circulating fatty acids that lead to oxidative stress, a key pro-inflammatory hallmark of NASH [20].

Two cellular organelles, mitochondria and peroxisomes, are involved in free radical production. An abnormal ROS amount is generated in mitochondria as a consequence of increased β-oxidation due to the increased influx of circulating fatty acids into hepatocytes. Peroxisomes are also involved in ROS production through the breakdown of long-chain fatty acids, very-long-chain fatty acids and branched fatty acids. Furthermore, peroxisomes are also depots of some antioxidant enzymes (catalases and superoxidase) which neutralize hydrogen superoxide and superoxide anions [21]. In normal conditions, the dual role of peroxisomes as ROS producers and ROS scavengers is balanced. High levels of ROS generate disequilibrium in relation to antioxidant defense mechanisms, resulting in oxidative stress and mitochondrial and peroxisomal dysfunction that may exacerbate pro-inflammatory events of NASH [22,23,24,25].

It remains unknown whether the increased steatosis susceptibility in mice lacking PPARα depends on PPARα activity only in hepatocytes or also in other organs. The focus of the present review is the role of PPARα in liver metabolism and in NASH pathogenesis.

## 2. Main Structural and Functional Features 

**Figure 1:**
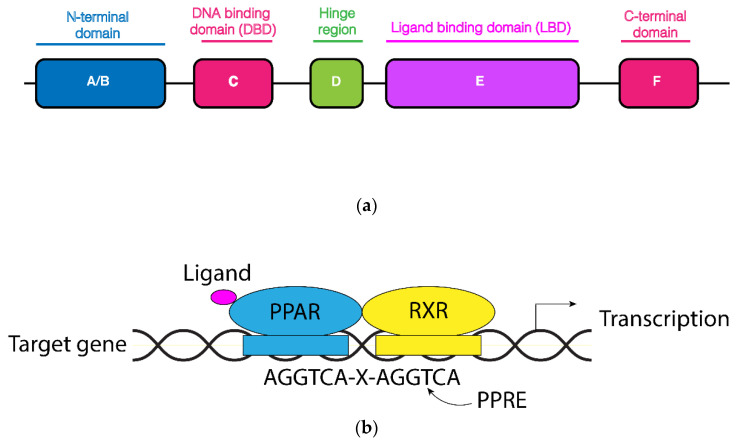
Structural organization of PPAR nuclear receptors and PPRE binding sites. (**a**) The N-terminal A/B domain encompasses a ligand-independent activation function 1. The C-terminal ligand-binding region includes the ligand-dependent activation function 2 interface. (**b**) PPAR heterodimerizes with RXR and binds to PPRE binding sites in the promoter region of the target genes. Likely, with a coactivator complex, this may associate and foster the transcription.

Among the peroxisome-proliferator-activated receptors, PPARα is the only member that is activated by peroxisome proliferators [26]. Peroxisome proliferators encompass different compounds that cause peroxisome proliferation and liver cancer in mice. The PPAR subfamily belongs to the larger family of nuclear receptors which includes receptors for fat soluble vitamins, steroid hormones and sterols [27]. The structure of nuclear receptors consists of shared domains: a variable N-terminal region (A/B domain) involved in transcriptional activation, a central and conserved DNA-binding domain containing a zinc-twist structure, a flexible hinge region and a C-terminal ligand-binding region (Figure 1a) [28].

PPARs bind to DNA as a heterodimer with the Retinoid X Receptor (RXR), and together they recognize the PPAR response element (PPRE) within the promoter of the target genes. Usually, the PPREs consist of a direct repeat of the consensus hexanucleotide AGGTCA spaced by a single nucleotide (Figure 1b). Heterodimer formation can also be induced by agonist ligands for PPAR which promote the recruitment of histone acetylase and histone acetyltransferase activity, respectively, necessary for the assembly of the transcription initiation complex. Each PPAR exhibits a different tissue expression and a specific ligand-binding affinity. There are classical PPARα ligands such as fenofibrate and clofibrate and PPARβ/δ ligands as GW501516, while the rosiglitazone is a long-established PPARγ ligand. Recently, dual-PPAR and pan-PPAR agonists have been under investigation. Following ligand attachment, PPAR translocates to the nucleus where it heterodimerizes with RXR [29]. Therefore, the knowledge of which PPAR member regulates a specific gene depends on the combination of two conditions: in which tissue(s) a gene is expressed and to which PPAR ligand(s) the gene is responsive. Likely, the same gene can be regulated by different PPARS in different tissues. PPARs may also bind to repressor proteins, thus reducing gene transcription [30]. Most of the ligands for PPARs encompass different types of (dietary) fatty acids and fatty-acid-derived compounds, including various eicosanoids [31]. PPARs are the molecular target of different classes of drugs used in the treatment of diabetes and dyslipidemia. Although now PPARα is present in many tissues, most of our knowledge derives from the studies in the liver under normal and pathological conditions.

## 3. PPARα and Liver Metabolism

The liver regulates the homeostasis of glucose and lipids in the body as well as the energy metabolism, both in fasting and in post-prandial conditions. As the metabolic center of regulation of blood glucose levels, the liver addresses glucose mainly towards the glycolysis and glycogen synthesis in postprandial conditions and has the task of releasing glucose from glycogenolysis and gluconeogenesis in fasting condition [32,33]. The liver also ensures the regulation of lipid metabolism by accumulating fats and cholesterol coming from the diet and converting excesses of fatty acids in ketone bodies to supply energy for extrahepatic tissues, such as brain and skeletal muscle, in fasting conditions [32]. All these metabolic processes are strictly regulated by different master regulators which trace the fate of glucose and lipids throughout the body [34,35]. In this context, the switch between the synthesis and degradation of fatty acids is a highly coordinated process. In the liver, this process is regulated by different transcriptional factors and nuclear receptors, among which PPARα is the main transcriptional regulator of genes involved in lipid metabolism and acts as a nutritional sensor by modulating the rate of biosynthesis and catabolism of free fatty acids (FFAs).

**Figure 2:**
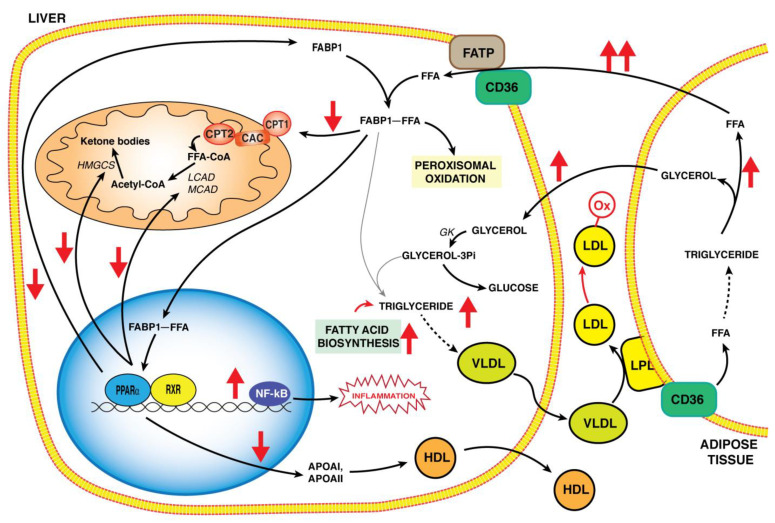
A summary of PPARα’s role in hepatic lipid metabolism pathways and alterations in the pathogenesis of NASH (red arrows). In hepatocytes, PPARα participates in the regulation of gene expression involved in lipid metabolic pathways such as FABP1, controlling trafficking, delivery and storage of FFA, or LCAD and MCAD, involved in mitochondrial β-oxidation. Liver FFA accumulation, derived also by mobilization of triglycerides from adipose tissue, together with a decrease in PPARα activation, leads to the alteration of liver lipid homeostasis by causing lipotoxicity in NASH (red arrows). Dysregulation of the lipoprotein metabolism causes a decrease of HDL and the formation of LDL and oxidized LDL that contributes to foam cell formation and finally to atherosclerosis. Abbreviations: FAPT: fatty acid transport protein; CD36: fatty acid translocase; FABP1: fatty acid binding protein-1; FFA: free fatty acid; CPT1 and CPT2: carnitine palmitoyltransferases 1 and 2; CAC: carnitine acylcarnitine carrier; MCAD: medium-chain acyl-CoA dehydrogenase; LCAD: long-chain acyl-CoA dehydrogenase;: HMGCS: 3-hydroxy-3-methylglutaryl-CoA synthase; PPARα: peroxisome proliferator activated receptor α; RXR: Retinoid X receptor; LPL: lipoprotein lipase; VLDL: very-low-density Lipoprotein; LDL: low-density lipoprotein; LDL-ox: oxidized low-density lipoprotein.

FFAs are transported in the hepatocytes through a fatty acid transport protein (FATP) and a fatty acid translocase (FAT), CD36, which facilitate the uptake of long-chain fatty acids (LCFA) [36,37]. Once inside the hepatocytes, FFAs bind to fatty acid binding protein-1 (FABP-1) [38] and are sent to their fate by depending on nutritional conditions. In particular, FFAs undergo the peroxisomal β-oxidation, or are transported into mitochondria by the shuttle of carnitine-palmitoyl transferase/carnitine-acyl carnitine carrier (CPT/CAC) going through β-oxidation (Figure 2). Alternatively, FFAs are addressed to the endoplasmic reticulum for lipid biosynthesis or to the nucleus as PPARα ligands to regulate the transcription of genes protecting the liver from lipotoxicity [17]. Both CD36 and FABP-1 are positively regulated by PPARα, and in particular, PPRE elements were identified at the FABP-1 gene promoter [39]. FABP-1 is an abundant protein localized in the cytoplasm of hepatocytes and is overexpressed during fasting. In fact, it transports LCFA towards mitochondria and interacts specifically with CPT1, which is the rate-limiting step in mitochondrial FFA undergoes β-oxidation [40].

Interestingly, PPARα regulates the expression of some genes for mitochondrial FFA β-oxidation, such as medium-chain acyl-CoA dehydrogenase (MCAD) or long-chain acyl-CoA dehydrogenase (LCAD) [41,42]. Additionally, FABP-1 transports LCFA to the nucleus and binds to PPARα by regulating the transcriptional expression of the LCFA metabolism genes as well as participates to its own transcriptional regulation as a closed circle to increase the FFA metabolism in fasting conditions. Moreover, ligand-activated PPARα upregulates the expression of ketone bodies’ biosynthesis genes to the same level as that of mitochondrial 3-hydroxy-3-methylglutaryl-CoA synthase (HMGCS), which harbors a PPRE sequence localized at −104/−92 bp in its promoter [43]. HMGCS is a key ketogenic enzyme which catalyzes the condensation of acetyl-CoA and acetoacetyl-CoA to generate 3-hydroxy-3-methylglutaryl-CoA (HMG-CoA), which in turn produces acetoacetate, the first ketone body. Therefore, PPARα is considered as a critical factor for ketogenesis and a PPARα-null mouse liver presents an impaired expression of HMGCS gene [43]. The stimulated ketogenesis by liver PPARα protects from liver lipotoxicity by decreasing lipid peroxidation and ROS production via increased β-hydroxybutyrate levels. Ketone bodies play a role as modulators of cellular signaling by inhibiting histone deacetylase activity (HDAC). The HDAC-mediated deacetylation leads to histone hypoacetylation, which is associated with gene transcriptional repression, while HDAC inhibition alters gene expression, in particular for those genes involved in cell survival and oxidative stress responses [44,45]. In this context, PPARα-mediated ketogenesis ensures metabolic flexibility during fasting as well as cytoprotection in physiological and pathological conditions.

PPARα activity also affects the lipoprotein metabolism by leading to a reduction in both very-low-density lipoproteins (VLDL) synthesized in the liver and low-density lipoproteins (LDL) and to an increase of high-density lipoproteins (HDL) (Figure 2) [43]. In particular, in rat liver, PPARα ligands act by increasing lipoprotein lipase activity (LPL), which catalyzes the hydrolysis of plasma triglycerides contained in lipoproteins. The LPL gene also harbors a PPRE motif located in its promoter, and its expression is directly regulated by PPARα [46]. Notably, this mechanism contributes to hypotriglyceridemic effects in vivo. Still, the higher plasma levels of HDL are related to the increased gene expression of apolipoproteins A-I (APOAI) and A-II (APOAII), the major proteins of HDL. Both genes encompass PPRE motifs in their promoters [43] and PPARα-gene expression regulation leads to an increase in plasma HDL levels. Interestingly, this PPARα regulation should have a hypocolesterolemic effect in vivo.

In light of these considerations, PPARα promotes the transport and oxidation of fatty acid and ketogenesis, pointing out the role of PPARα as a master regulator of the hepatic lipid metabolism in fasting conditions. Notably, PPARα also affects, directly and indirectly, lipogenesis, the metabolic process dependent upon the nutritional conditions and activated by a carbohydrate-rich diet. The hepatic lipogenesis is under the control of the sterol regulatory element binding protein 1c (SREBP-1c) transcriptional factor [47,48], whose hepatic activation leads to the control of lipogenic genes such as fatty acid synthase (FAS) and acetyl-CoA carboxylase (ACC) in a diet-related condition. SREBP-1c activity is affected by PPARα agonists, which bind SREBP-1c in human hepatocytes [49]. PPARα is considered an activator of the SREBP1c promoter in vivo, by direct interaction or by a crosstalk between PPARα and the liver X receptor (LXR). In effect, the LXR promoter region encompasses a PPRE element and is activated by PPARα. In turn, the SREBP1c promoter contains LXRE active sites being activated by LXR [49]. Furthermore, the levels of SREBP1c mRNA are decreased in PPARα-null mice livers [50] and are increased in mice treated with PPARα agonists [51]. These results suggest that PPARα participates in the regulation of SREBP1c by sustaining the switch between fasting and post-prandial conditions [49]. Thus, PPARα participates in the regulation of fatty acid biosynthesis in different conditions: first of all, in a carbohydrate-rich diet; second to compensate for the increased fatty acid oxidation in fasting conditions, as a part of the triglyceride/fatty acid cycle.

The function of PPARα should be considered as a short and long regulation mode. The short regulation mode acts early in fasting condition to address FFAs towards oxidation for cellular needs. The long regulation mode may contribute to avoiding a higher oxidation of FFAs and a higher production of ketone bodies by regulating the lipoprotein and lipogenesis metabolisms through PPARα or PPARα ligands. The direction of metabolic activation via PPARα should depend on liver FFA levels. When the FFA concentration exceeds the oxidation rate and some metabolic products, such as ketone bodies, are accumulated, PPARα induces lipogenesis. On the contrary, when liver fatty acid uptake increases or liver fatty acid efflux decreases, PPARα activation prevents the accumulation of fatty acid by stimulating FFA degradation. In this context, we can hypothesize that PPARα takes place in the triglyceride/fatty acid cycle to ensure the hepatic homeostasis of fatty acid metabolism.

## 4. PPARα as a Modulator of Inflammatory Pathways

PPARα, as well as the other members of the PPAR family, modulates different inflammatory signaling pathways through negative transcriptional regulation [52,53]. This mechanism is termed transrepression and consists of the binding of PPARs to the PPREs of target genes [53,54]. In detail, PPARα negatively modulates the expression of genes responsible for inflammation (i.e., adhesion molecules, extracellular matrix proteins and cytokines) by inhibiting the transcriptional activities of pro-inflammatory transcription factors, such as the nuclear factor kappa B (NF-κB), activator protein 1 (AP-1) and signal transducer and activator of transcription (STAT) [55].

NF-κB can be inactivated by the direct binding of PPARα to subunit p65 of NF-κB [56]. Other inhibitory mechanisms are possible. PPARα can bind to p300, the histone acetyltransferase responsible for the acetylation of Lys310 on subunit p65 NF-κB [57,58,59], a post-translational modification fundamental for NF-κB activation [60]. Moreover, the activation of PPARα promotes the expression and activity of Sirtuin 1 (SIRT1), which inhibits NF-κB by deacetylation [61,62]. PPARα can indirectly regulate NF-κB by the upregulation of IκBα, the NF-κB inhibitor, since the promoter of the gene encoding for IκBα is under PPARα’s transcriptional control [63,64].

PPARα binds to c-Jun and consequently inhibits AP-1 activation and DNA-binding activity [56]. The proinflammatory genes, which have an AP-1-binding site in their promoter, such as PTGS2 encoding for cyclooxygenase 2 (COX-2), have inhibited results [65]. The activities of STAT1 and STAT5b are disrupted by PPARα [66,67,68]. Besides the direct effect on transcription factors, PPARα increases the expression of antioxidant enzymes such as catalase, superoxide dismutase and heme oxygenase-1, which reduces ROS levels [69,70]. Furthermore, in PPARα-deficient mice, increased levels of IL13 and GATA-3 have been found [71]. GATA-3 is a transcription factor selectively expressed in murine during Th2 polarization and controls genes encoding for Th2 cytokines, in particular IL-13 and IL-5 [72]. 

Interestingly, pro-inflammatory effects and involvement in tissue repair have also been ascribed to PPARα. Zhang and Ward have demonstrated that WY-14 643 [PPARα activator, 4-cholro-6-(2.3-xylidino)-2-pyrimidinaylthio acetic acid], a potent PPARα agonist, stimulates the secretion of the pro-inflammatory cytokines IL-1β, IL-6, IL-8 and TNFα in ocular cells [73]. Additionally, WY-14 643 evokes proangiogenic responses [73]. Oxidized phospholipids and lipoproteins promote monocyte chemoattractant protein 1 (MCP-1) and IL-8 expression in a PPARα-dependent manner in human aortic endothelial cells and ocular cells [74]. Moreover, fenofibrate stimulates a higher LPS-induced TNF release in endotoxemia [75]. The treatment of murine macrophage cell lines with the two natural PPARα ligands leukotriene B4 and 8(S)-hydroxyeicosatetraenoic acid stimulated the expression and activity of nitric oxide synthase, resulting in a pro-inflammatory phenotype [76]. 

In light of the above, it is possible to conclude that the activation of PPARα could generate different responses according to the inflammatory context.

## 5. PPARα Expression in NASH Liver 

Though a healthy liver does not accumulate lipids, the liver plays regulatory roles in lipid anabolism by exporting fatty acids to peripheral organs, including white adipose tissue for energy storage, and in lipid catabolism by mobilizing them from the adipose tissue in a fasting state. PPARα involvement in the activation of lipid metabolism genes is well known [7]. PPARα activation induces expression of a wide range of genes involved in all steps of lipid metabolism: transport, binding, uptake, synthesis, mitochondrial and peroxisomal degradation, storage, lipoproteins metabolism and ketogenesis in fasting [43]. 

PPARα is highly expressed at similarly high levels in human and rat liver [77]. A truncated, dysfunctional and less expressed isoform has also been found in human liver due to the introduction of a premature stop codon (exon 6 lacking) [78]. Interestingly, Thomas et al. showed that the truncated isoform can prevent inflammation in human liver cells [78]. This observation led to investigations into the link between PPARα abundance and NAFLD as well as NASH. An investigation performed on patients with suspected NAFLD (free of NASH) found no expression difference of PPARα between healthy subjects and NAFLD ones [79]. In another study performed on subjects with suspected NAFLD, including those with NASH, lower levels of PPARα were found in the liver of NASH patients [80]. Liver PPARα expression was negatively correlated with the presence of NASH and with the severity of steatosis, hepatocytes ballooning, the NASH activity score and fibrosis [80]. This correlation was also confirmed on analysis performed on immunohistochemical staining of PPARα in liver biopsies. Kersten et al. showed that PPARα staining intensity was lower in steatosis and much lower in the inflammatory state of NASH as compared to normal livers [81]. Park et al. reported that PPARα expression is also reduced in NASG induced by a methionine- and choline-deficient diet in mice together with a reduction in peroxisomal and mitochondrial fatty acid oxidation. Treatment with statins recovered the PPARα expression and fatty acid oxidation [82]. 

A crucial point concerns the reduction of PPARα in NASH. Experimental evidence from cultured liver cells highlighted that PPARα reduction could be related to the inflammatory process. Experiments performed on HepG2 cells showed this reduction is due to some inflammatory mediators, such as TNFα and IL-1β. Lim et al. showed that TNF-α suppresses PPARα mRNA expression in a dose- and time-dependent manner at the transcriptional level by enhancing the activity of canonical NF-κB signaling pathways [83]. Reduction of PPARα can also be due to the involvement of IL-6 at the level of gene transcription through the activation of a CCAAT/enhancer-binding protein, as uncovered by Chew et al. [84]. All these data suggest a strict correlation between inflammation, PPARα and NASH onset. 

## 6. Role of PPARα in the Pathogenesis of NASH

NASH is characterized by liver fat accumulation, insulin resistance as well as inflammatory conditions. PPARα is a common factor to all these processes [85]. The hepatic triglyceride accumulation is due to the imbalance of different events: an excess of dietary FFAs, an increase in liver lipogenesis and a decrease in liver FFA oxidation and VLDL synthesis (Figure 2) [86]. All these metabolic pathways, i.e., uptake, oxidation, lipogenesis and export, are regulated by PPARα and reflect the alteration on liver lipid homeostasis. In mice models with PPARα gene deletion, hepatic steatosis is a characteristic trait in fasting or in fat-rich diets, confirming the involvement of PPARα in these processes [87]. 

In NAFLD and in NASH, the increased plasma FFA levels derive from lipolysis of the adipose tissue, which represents the main source of accumulation of triglyceride in the liver [88]. This increase consequently causes lipotoxicity, insulin resistance, oxidative stress and inflammation, which are players in NAFLD and NASH and take a place in a closed circle in which everyone contributes to a positive feedback regulation. Lipotoxicity, due to lipid accumulation, causes mitochondrial dysfunction and impairs insulin signaling. Insulin resistance contributes to the alteration of the liver’s lipid metabolism by impairing the insulin-signaling-mediated lipolysis regulation [89]. Next to this, increased FFA uptake in the liver, insulin resistance and hyperinsulinemia induce lipogenesis in subjects with NAFLD and NASH by stimulating lipogenic enzymes via activation of SREBP-1c.

While in healthy subjects, lipogenesis is elevated in post-prandial conditions, in NAFLD subjects, lipogenesis contributes to 26% of the liver’s triglyceride content [90], and lipogenesis is already stimulated in fasting, confirming the upregulation of enzymes of liver lipogenesis also occurs in insulin resistance conditions. The expression of hepatic PPARα and PPARα-regulated genes related to fatty acid oxidation increases in high-fat diet conditions as an adaptive response [9,91]. In NAFLD subjects, the expression of hepatic PPARα is decreased, and mice models with PPARα deletion show a high accumulation of triglycerides [92], confirming an involvement of PPARα in NAFLD and NASH progression. In this context, adiponectin, the hormone secreted by adipose tissue, also plays an interesting role, by activating liver PPARα and by stimulating FFA oxidation. In obesity as well as in insulin resistance conditions, an altered adiponectin signalling was observed and a lower adiponectin level was found in NAFLD [93].

The alteration of the liver’s lipid metabolism leads to an increase in plasma-free FA, triglycerides and oxidized LDL (ox-LDL), and consequently, an increase in lipotoxicity, oxidative stress and inflammation. A hallmark of NAFLD and NASH subjects is an inflammatory condition in which inflammatory cytokines and transcriptional factors of inflammatory response are upregulated in the liver. A decrease in PPARα function causes a skip of the inactivation check on NF-κB by inducing the production of pro-inflammatory cytokines in NAFLD and NASH inflammatory conditions [94].

In conclusion, PPARα activation takes a central place in the regulation of inflammation by leading to the inhibition of inflammatory factors. Therefore, the decrease in PPARα expression in NASH is crucial for a strong inflammatory response.

## 7. PPARα and Peroxisomes in NASH

Hepatocytes contain a larger number of peroxisomes that are also bigger sized as compared to other cell types. They are estimated to occupy 2% of the liver’s parenchymal volume. The metabolic pathways in hepatic peroxisomes are α- and β-oxidation and the synthesis of ether lipids. In the α-oxidation pathway, one carbon is removed from the branched chain fatty acid phytanic acid and from 2-hydroxylated fatty acids [95]. In the β-oxidation very long chain fatty acids (VLCFA), dicarboxylic fatty acids (DCA) and pristanic acids (formed from phytanic acids) are degraded. The anabolic role involves the formation of mature bile acids from cholesterol and the synthesis of a polyunsaturated fatty acid, docosahexaenoic acid [95]. Different steps of peroxisomal fatty acid metabolism are under the transcriptional control of PPARα [10]. As consequence, the altered expression of PPARα can significantly affect fatty acid oxidation and induce lipogenesis, resulting in an increase in fatty acid production, one of the factors for the progression of NASH. Drugs acting as PPARα agonists, such as fenofibrate, upregulate the expression of genes, resulting in a reduction in lipid accumulation in the liver while maintaining elevated ex novo lipogenesis. Furthermore, fenofibrate administration completely corrected high-fructose-induced glucose intolerance, hepatic steatosis and the impaired hepatic insulin signaling (pAkt and pGSK3β) [96]. 

**Figure 3:**
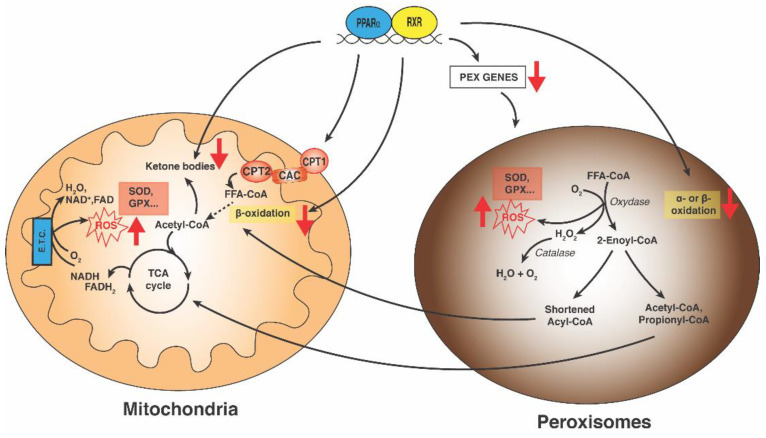
Functional role of PPARα in mitochondrial and peroxisomal lipid metabolism. PPARα regulates the expression of genes involved in peroxisomal α- and β-oxidation of very long chain fatty acids (VLCFA) and branched-chain fatty acids (BRCFA), together with genes of mitochondrial carnitine/acyl carnitine shuttle (CPT1/CAC/CPT2) and β-oxidation fatty acids. Both the mitochondrial and peroxisomal pathways lead to the production of ROS, and many antioxidant enzymes, among which catalase, glutathione peroxidase and superoxide dismutase contribute to ROS detoxification in both cellular compartments. Fat overload causes an elevated hepatic uptake of FFAs (Free Fatty Acids). In NASH, the altered mitochondrial and peroxisomal FA oxidation, together with a decrease in PPARα expression, lead to mitochondrial dysfunction, mtDNA and protein alteration, and a decrease in the electron transport chain, which causes an increase in ROS production. In turn, the dysregulation of peroxisomal biogenesis exacerbates the increase in oxidative stress. Abbreviation: PEX genes: genes of peroxisomal biogenesis factors; SOD: superoxide dismutase, GPX: glutathione peroxidase; ETC: electron transport chain; CPT1: carnitine palmitoyltransferase 1; CPT2: carnitine palmitoyltransferase 2; CAC: carnitine/acylcarnitine carrier.

Peroxisomes also contain detoxifying enzymes, catalase and superoxidase, aimed at the degradation of ROS produced in different oxidation reactions. Different Acyl-CoA oxidases, the rate-limiting enzymes of peroxisomal β-oxidation, generate H_2_O_2_. Acyl-CoA oxidase knock-out mice are defective in peroxisomal β-oxidation and develop hepatic steatosis [97]. H_2_O_2_ is also produced in other oxidative reactions catalyzed by D-amino acid oxidases, 2-hydroxy acid oxidases (HAO), L-pipecolate oxidase and alanine glyoxylate aminotransferase [98]. Thus, peroxisomes are faced with two situations: on the one hand, the production of ROS is mainly derived from fatty acid oxidation (Figure 3), on the other hand, there is the need to neutralize them. Increased levels of circulating fatty acids and increased fatty acid oxidation generate a high amount of ROS to be neutralized, suggesting that the liver depends much more on peroxisome for a safe disposal of ROS in NASH [99]. Scavenging of ROS is crucial to prevent oxidative stress. Steatosis liver induced by a fatty diet in catalase knock-out mice causes an accelerated onset of oxidative stress and inflammation [100]. 

Defects in genes involved in the biogenesis of peroxisomes may lead to hepatic steatosis, steatohepatitis and fibrosis. Different proteins are necessary for new mature peroxisomes. These proteins, named peroxins (PEX), are required for the import of peroxisomal matrix proteins from the cytosol, the formation of the peroxisomal membrane and the fission of existing peroxisomes [101]. Currently, mutations in 14 PEX genes are known to cause peroxisome biogenesis disorders, some of which result in hepatic steatosis, steatohepatitis and fibrosis [102]. For example, mice lacking Pex5 develop steatosis [103]. Furthermore, upregulation of peroxisomal biogenesis protects against the onset of steatosis induced in mice fed fatty diets [104]. All these findings provide evidence on the role of PPARα-mediated peroxisome functionality in the onset of steatosis and NASH. 

## 8. PPARα and Mitochondria in NASH

Similarly to peroxisomes, mitochondria are dynamic organelles that continuously adapt their number, morphology and function to prevailing environmental conditions [105]. Their role in the production of ATP, β-oxidation of fatty acids, ketone body biosynthesis and iron–sulphur cluster synthesis is well known [106]. Many enzymatic steps of fatty acid oxidation, from the mitochondrial uptake and subsequent oxidative breakdown of acyl-CoAs to acetyl-CoA, are regulated by PPARα [10]. Specifically, PPARα stimulates acyl-CoA import into the mitochondria by upregulating expression of carnitine cycle members: carnitine palmitoyl-transferases (CPT1 and CPT2), and the acyl-carnitine translocase (Slc25a20) (Figure 3) [107,108,109]. Likewise, genes involved in the cellular uptake and biosynthesis of carnitine, as well as OCTN2, are 4-trimethylaminobutyraldehyde dehydrogenase, encoded by the SLC22A5 gene, TMABA-DH, encoded by the ALDH9A1 gene and γ-butyrobetaine dioxygenase, encoded by the BBOX1 gene, all of which are PPARα-regulated [42,110,111]; PPARα null mice also show a decreased expression of carnitine biosynthetic genes [112]. Moreover, β-oxidation acyl-CoA dehydrogenases, such as LCAD and MCAD and subsequent chain shortening reactions catalyzed by the mitochondrial trifunctional enzyme (Hydroxyacyl-CoA Dehydrogenase Trifunctional Multienzyme Complex Subunit Alpha, encoded by HADHA gene and Subunit Beta, encoded by HADHB gene), are PPARα-induced reactions [41,42]. Other enzymes, such as hydroxyacyl-CoA dehydrogenase (HADH), acetyl-CoA acyltransferase 2 (ACAA2) and those required to convert unsaturated and 2-methylated acyl-CoAs into intermediates of β-oxidation (Enoyl-CoA Delta Isomerase 1 -Eci1-, Enoyl-CoA Delta Isomerase 2 (Eci2), 2,4-Dienoyl-CoA Reductase 1 -Decr1-, Hydroxysteroid 17-Beta Dehydrogenase 10 -Hsd17b10-) are PPARα targets [41,113]. 

In fasting conditions, PPARα also regulates the expression of four genes of the ketogenesis as well as the transcription of acetyl-CoA acetyltransferase 1, encoded by ACAT1, 3-hydroxy-3-methylglutaryl-CoA synthase 2, encoded by HMGCS2, 3-hydroxy-3-methylglutaryl-CoA lyase, encoded by HMGCL, and 3-hydroxybutyrate dehydrogenase 1, encoded by BDH1, which is mediated by PPARα [114]. High-fat diets and dysregulation of the lipid metabolism result in the accumulation of hepatic FFA and triglycerides, which enhances mitochondrial fatty acid oxidation. However, if the accumulation of fat is continuous and prolonged, the adaptation of the mitochondria is no longer sufficient to prevent lipotoxicity of the fatty acid depots. As a consequence, hepatic steatosis develops gradually, resulting in the onset of severe non-alcoholic steatohepatitis. Moreover, any impairment of PPARα expression and activity impacts mitochondrial fatty acid oxidation.

Mitochondria are considered as the major source of ROS within cells, in particular at the level of complex I and III of the respiratory chain (Figure 3). However, other sites within mitochondria can produce ROS [115]. Like peroxisomes, mitochondria contain antioxidant defense systems: superoxide dismutase 1/2 (SOD1/SOD2) enzymes, glutathione/glutaredoxin, thioredoxin/peroxiredoxin systems and low-molecular-weight antioxidants such as CoA, ubiquinol and vitamin C [116].

A redox interplay between mitochondria and peroxisome exists since defects in peroxisome biogenesis, peroxisomal β-oxidation or catalase activity induce mitochondria oxidative stress in the liver and other organs. 

## 9. Targeting PPARα as Therapy for NASH

Due to the lack of FDA-approved medications, lifestyle modifications, in particular weight loss, are currently the first line of treatment for NASH. Because of their key roles as lipid regulators and inducers of anti-inflammatory responses, transcription factor PPARs represent exemplary targets in NAFLD therapy. However, to date, targeting only PPARα has had limited success. The fibrates which act as PPARα agonists, such as Fenofibrate and Gemfibrozil, improve some symptomatic aspects of NASH such as liver function, lipid profile and insulin sensitivity, but do not influence the histopathology and have numerous side effects (e.g., impaired kidney function, increase in serum creatinine and homocysteine) [117,118,119,120,121]. Furthermore, the combination of fibrates with some drugs (e.g., gemfibrozil and cerivastatin) increases the incidence of adverse effects, such as rhabdomyolysis [122]. 

Pemafibrate, a novel selective PPARα modulator, amends NASH histological features in mice [123] and serum alanine aminotransferase (ALT) in NASH patients with dyslipidemia [124]. A double-blind, placebo-controlled, randomized multicenter phase II trial (NCT03350165) has just shown that pemafibrate significantly reduces liver stiffness, even if it does not affect liver fat content [125]. 

The simultaneous activation of more than one PPAR by using dual- and pan-PPAR agonists is becoming a promising strategy for NAFLD and NASH therapy [126,127]. GOLDEN-505, a phase IIb study aimed at evaluating the efficacy and safety of Elafibranor, a dual PPARα/δ agonist, in patients with NASH, highlighted the lower NAFLD activity score, resolution of NASH without fibrosis, reduction in inflammatory markers, improvement in insulin sensitivity, glucose homeostasis and lipid metabolism [128]. Unfortunately, the phase III trial, RESOLVE-IT, focused on comparison between elafibranor and the placebo in 2157 NASH patients (NCT02704403), was prematurely terminated because of limited efficacy at the time of interim analysis. 

Saroglitazar, a dual-PPARα/γ agonist, in a randomized, double-blind, placebo-controlled study (EVIDENCES IV, NCT03061721), significantly improved liver function, serum ALT, insulin resistance and atherogenic dyslipidemia [129]. Two clinical trials designed to evaluate the safety, tolerability and efficacy of Saroglitazar in women with polycystic ovary syndrome (NCT03617263) and in liver transplant recipients with NAFLD (NCT03639623) are running. 

Lobeglitazone is another dual-PPARα/γ agonist able to dampen intrahepatic fat content and ameliorate liver enzymes together with glycemic and lipid profiles in type 2 diabetes patients with NAFLD [130], as it has been demonstrated in a Phase IV trial (NCT02285205). 

Lanifibranor is a pan-PPAR agonist that regulates different metabolic, inflammatory and fibrogenic pathways with a key role in the pathogenesis of NASH. For these abilities, it was investigated for the treatment of NASH in a double-blind, randomized, placebo-controlled phase II trial (NCT03008070). The results indicated lanifibranor for the resolution of NASH: liver enzyme levels decreased and the majority of lipid, inflammatory and fibrosis biomarkers improved. Side effects such as diarrhea, nausea, peripheral edema, anemi, and weight gain occurred more frequently with lanifibranor than with the placebo at a rate less than 5% [131]. Another clinical trial aimed at establishing the safety, efficacy, and mechanism of action of lanifibranor in patients with type 2 diabetes and NAFLD is ongoing (NCT03459079). 

In conclusion, the treatment of NASH is a tall task. Therefore, it seems that agonists able to activate PPARα together with other PPAR members are promising drugs to resolve NASH.

## 10. Conclusions

This review describes the role of PPARα in governing lipid metabolism in the liver and its alterations in the pathogenesis of NASH (Figure 2). Evidence highlights that PPARα is differently expressed in NAFLD and NASH livers. The reduced PPARα expression in NASH is crucial for a strong inflammatory response. Mitochondrial and peroxisomal functions are compromised in NASH and may contribute to the progression of the disease. This finding may also be due to the reduced expression of PPARα. Although the decreased PPARα expression in NASH seems due to the suppressive effect of some cytokines, the sequence of events observed in NAFLD and NASH progression is not completely clear. This is a limit to the development of efficient therapies. At present, therapeutic approaches are focused on antioxidative compounds (phenolic compounds) to counteract ROS production. In addition, PPARα agonists, such as fibrates, are used to increase its expression. However, these compounds are not efficient at fighting the typical features of advanced NAFLD, such as NASH. Thus, future studies are required to understand the sequence of events in NAFLD and NASH progression and the specific role played by PPARα. This will lead to considerations of PPARα as a better molecular target, alone or in combination with other PPAR factors, in the treatment of NASH and other liver inflammatory diseases. In addition, a better understanding of PPARα regulation by different signals (i.e., nutrition, drugs, etc.) will allow for the designing of more specific and strong pharmacological therapies. 

## Data Availability

Not applicable.
